# Hands as Sex Cues: Sensitivity Measures, Male Bias Measures, and Implications for Sex Perception Mechanisms

**DOI:** 10.1371/journal.pone.0091032

**Published:** 2014-03-06

**Authors:** Justin Gaetano, Rick van der Zwan, Duncan Blair, Anna Brooks

**Affiliations:** 1 Cognitive Neuroscience Research Cluster, Southern Cross University, Coffs Harbour, Australia; College of Mechatronics and Automation, National University of Defense Technology, China

## Abstract

Sex perceptions, or more particularly, sex discriminations and sex categorisations, are high-value social behaviours. They mediate almost all inter-personal interactions. The two experiments reported here had the aim of exploring some of the basic characteristics of the processes giving rise to sex perceptions. Experiment 1 confirmed that human hands can be used as a cue to an individual’s sex even when colour and texture cues are removed and presentations are brief. Experiment 1 also showed that when hands are sexually ambiguous observers tend to classify them as male more often than female. Experiment 2 showed that “male bias” arises not from sensitivity differences but from differences in response biases. Observers are conservative in their judgements of targets as female but liberal in their judgements of targets as male. These data, combined with earlier reports, suggest the existence of a sex-perception space that is cue-invariant.

## Introduction

The ability to quickly and accurately to discriminate whether another individual is female or male is considered one of only a few automatic and thus fundamental aspects of person perception [1]. The development of perceptual processes capable of discriminating, at a distance, another’s sex conveys considerable advantage by priming a range of different behaviours [2]–[6]. Of course, for an observer to be able to discriminate another’s sex, there must exist cues that are sexually dimorphic and predictably so. The obvious candidates are the primary sex cues. In many animals, however, body posture or body morphology often conceals the genitalia from view. In upright humans the primary sex cues are not concealed by morphology but have nonetheless typically been obscured, probably since humans first took to wearing clothes some 170 000 years ago [7]. There are, however, other sexually dimorphic visual [8]–[12], auditory [13], and olfactory [14] cues that humans can exploit, either alone [15]–[19] or in combination [2], [20]–[23] to perform the task of sex discrimination. We do so, most of the time, with remarkable precision.

Of the modalities mentioned, the one for which the most extensive sex perception literature has developed is vision. Studies within that literature use either whole-body or partial-body representations to systematically explore the processes mediating sex perceptions. In the case of the whole-body literature, studies have generally focussed on sexually dimorphic structural [24], [25] and/or kinematic cues to sex [18], [26]–[29]. By comparison, studies of partial-body sex perceptions employ, almost exclusively, images of the face as stimuli [16], [19], [30], [31], although hands also have been used [32]. No matter which stimulus type is used, there are some interesting convergent findings. For example, whole-body representations [18], [33], faces [34]–[36], and hands [32] all can be used to elicit sex aftereffects. For whole-body and for face stimuli there also are reliable reports of a so-called *male bias*, a tendency to report normally dimorphic stimuli as looking male (rather than female) when the dimorphic cues are ambiguous [17], [18], [29], [37]–[42].

That pattern of convergence suggests the mechanisms processing sex cues might operate in such a way as to give rise to a multi-dimensional sex perception-space, analogous to the space already proposed for face perceptions [17], [43]–[45]. In such a space cues to “femaleness” and cues to “maleness” would converge, independent of their source. Rather than only cue-dependent, or perhaps sense-dependent mechanisms mediating sex perceptions, such a space would be adaptive [46], taking into account all available and relevant information.

In addition to the apparent ubiquity of the male bias, the observations that object-to-face [47], body-to-face [48], and foot-fall-to-gait [23] sex aftereffects manifest certainly support that idea. There is some evidence, however, that the processes handling sex cues, similar or not, are independent of each other. For example, Kovács et al. [32] found sex aftereffects only when adaptor and test stimuli were the same body part – faces *or* hands. No effects were observed when adaptor and test respectively depicted faces *and* hands (or hands and faces). That is evidence against convergent processing, and suggests “femaleness” and “maleness” are stimulus-dependent.

With that in mind, the experiments reported here were designed to begin to explore the proposal that there exists a multi-dimensional sex-perception space built around all available sexually dimorphic cues. Such a space would be supported, in the cortex, by higher-order mechanisms onto which sensory processes converge. Those mechanisms, like any in the cortex, will give rise to predictable behaviours characteristic of sex perceptions. Thus, and in particular, it was hypothesised that if such a space exists, the male bias reported already for full-body and face perceptions will manifest also for hands. In comparison to the vast literature describing face perceptions the literature around hands is pauce. The two experiments reported here unpack more fully observers’ sensitivity to hand-based sex cues and, in that context, the perceptual processes giving rise to that sensitivity.

## Methods

### Ethics Statement

All observers gave written, informed consent prior to participating in the study. Ethical approval for this study was granted by the Human Research Ethics Committee of Southern Cross University (ECN-10-115). This study complies with the ethical standards specified by the Declaration of Helsinki.

### Observers and Apparatus

Originally, 12 experienced psychophysical observers (6 female, 6 male) participated in this study, but data corresponding to 1 male were discarded due to apparent response confusion. All trials were conducted in a light- and sound-attenuated psychophysics laboratory using a computer monitor linearised for luminance. Responses were recorded via key-press on a standard computer keyboard. Stimuli were presented using ePrime software (*Psychology Software Tools*, 2011).

### Stimuli

Digital photographs were taken of both the dorsal and palmar surfaces of 15 female and 15 male hands. Those photographs formed the basis of the stimulus sets used in these experiments. Hand models were excluded from participation as experimental observers. Hand posture was standardised (fingers together with thumb held close to the first finger). All adornments (bracelets, rings, and so on) were removed before photographs were taken. For standardisation purposes, each hand was placed on a grid of 1×1 cm squares, such that the middle finger was aligned both with the centre axis of the grid and the length of the forearm.

Across images, orientation was normalised and sex-stereotypic cues (e.g. long fingernails, tattoos, and scars) were removed digitally via Photoshop CS4 (*Adobe Systems*, 2008). To control for global size-based heuristics (i.e. observers might base their categorisations of sex *solely* on the appearance of each hand as ‘small’ or ‘large’) the absolute size of the stimuli were controlled. Each exemplar was scaled to a standard size whilst simultaneously maintaining its natural height-to-width proportions [16], [17].

Absolute size scaling was achieved using two techniques: In one stimulus set, the overall size (as indexed by total pixel count) was *reduced* to that of the smallest hand (44,693 px, ±10%); in the other, it was *enlarged* to that of the largest hand (89,394 px, ±10%). In the reduced set, the number of pixels difference between each standardised exemplar and the median was calculated separately for female and male hand stimuli. A mixed Analysis of Variance (ANOVA) allowed us to confirm that the variance across standardised pixel counts was the same irrespective of stimulus sex (*F*_1,28_ = 1.36, *p* = .253), stimulus surface (palmar, dorsal; *F*_1,28_ = 0.34, *p* = .565), or the interaction between those two factors (*F*_1,28_ = 0.58, *p* = .453).

The purpose of employing both the enlargement and reduction method was to reduce the likelihood that artefacts associated with one or the other method could be used to explain any observed results. For instance, there is the risk that manipulating the size of a digital image might result in the visible loss of that image’s clarity or integrity, meaning the largest (or smallest) exemplars are potentially degraded the most when reduced (or enlarged) to a standard size.

**Figure 1:**
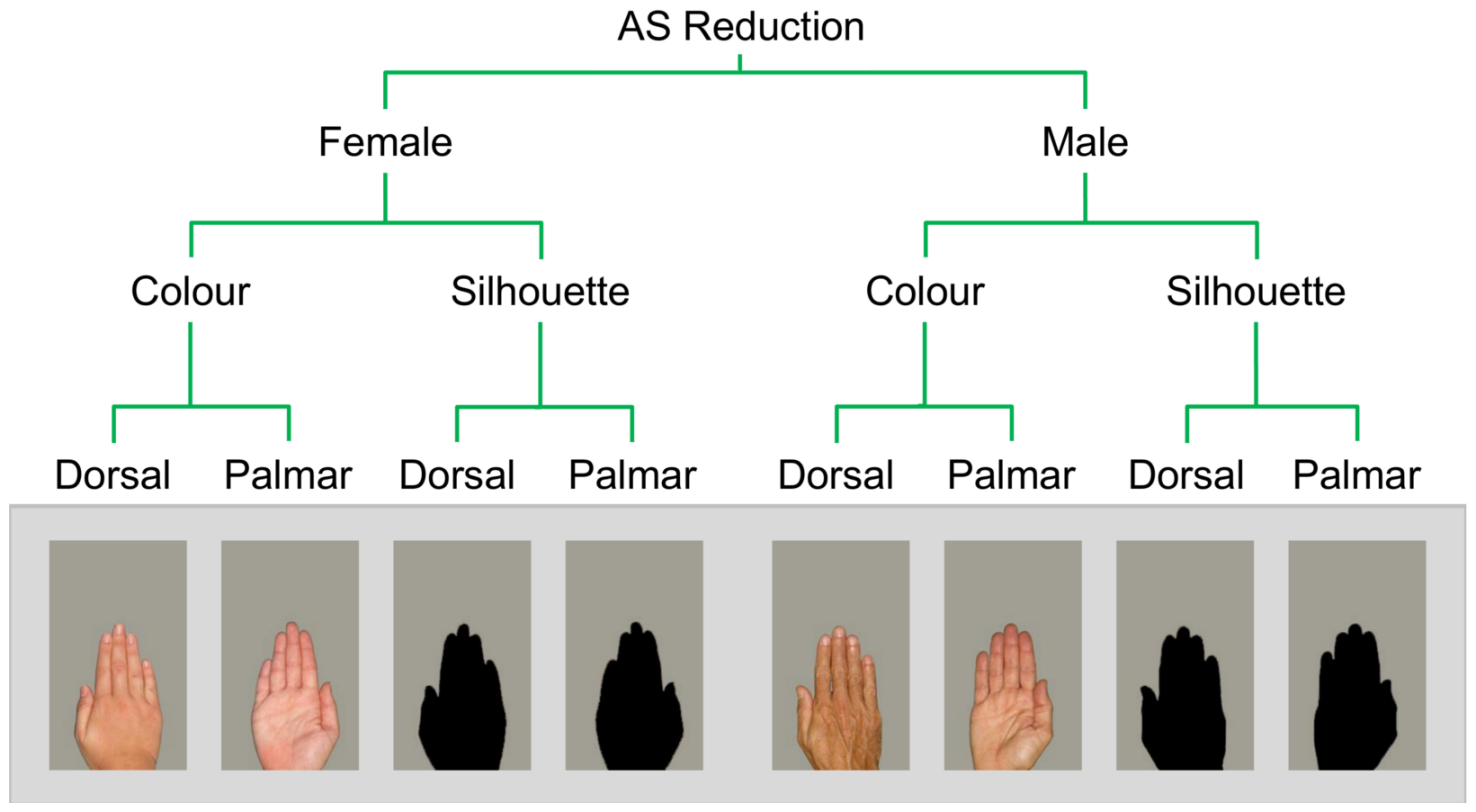
Stimulus exemplars used in these experiments. Hand stimuli were standardised for absolute size via a process of *reduction* (depicted) and *enlargement*. Within each stimulus condition 15 female and 15 male exemplars were represented. Each reduced and enlarged image had an absolute pixel count of 44,693 px and 89,394 px respectively (±10%).

Thus standardised for absolute size, stimuli were further manipulated in line with techniques from the face literature [17] to form two distinct sub-sets: one in which all hue and texture information was preserved (‘colour’ condition) and another in which those cues were removed (‘silhouette’ condition). In total then, the omnibus stimulus set comprised 240 images (30 hands [15 female, 15 male]×2 surfaces [dorsal, palmar]×2 conditions [colour, silhouette]×2 absolute size manipulations [reduced, enlarged]). Reduced stimulus exemplars are represented in Figure 1.

### Procedure

Both experiments used a two-alternative forced-choice design (2AFC) but differed with respect to response options and subsequent analyses. Each trial comprised in chronological order: a blank screen for 1000 ms, a stimulus presentation lasting 125 ms or 1000 ms, and a response screen (centred cross, +, on black background) that extinguished when either the observer made a response or 1000 ms had passed. The order in which blocks were presented was counterbalanced, as was the response key associated with each of the alternatives.

In Experiment 1, colour and silhouette images were presented in randomised order in blocks defined by presentation duration (125 ms or 1000 ms) and absolute size manipulation (reduced or enlarged). The observer’s task after each presentation was to indicate via key press whether the image represented a ‘female’ or ‘male’ hand. A subset of the stimuli used in Experiment 1 was employed for Experiment 2. To reduce the load on observers, and because no differences between the two different sized stimulus sets were observed (see Experiment 1: Results), the size-reduced stimulus set was arbitrarily selected for use. Similarly, because they were the most ambiguous stimuli used in Experiment 1, silhouette images were used in Experiment 2. Each image was presented twice across two target blocks (female/not female, male/not male) and two presentation durations (125 ms, 1000 ms) for a total of 240 trials.

### Analyses

Performances were averaged across viewing surface (palmer, dorsal) and then the potential mediating variables of absolute size and observer sex were analysed via omnibus ANOVA. Performances by each observer were then calculated as an average on all trials on each condition of interest (hue/texture [colour, silhouette], stimulus sex [female, male], and presentation duration [125 ms, 1000 ms]). Predictions were formally tested via planned orthogonal contrasts [49] designed to test each hypothesis in each experiment.

In Experiment 1, the dependent variable was the mean proportion of sex classification *errors* (i.e. pressing the ‘male’ key in response to a female hand or the ‘female’ key in response to a male hand). Data from Experiment 2 were analysed for two factors: sensitivity and bias, indexed by *d*-prime (*d*’) and criteria (*c*) scores, respectively [50]. Sensitivity here represents the ability of an observer to distinguish between target present (female- or male-) trials and target absent (female- or male-) trials. Sensitivity scores of zero indicate no sensitivity or chance performance: Observers cannot distinguish between target present and target absent trials. Similarly, increasingly positive *d*’ scores indicate increased sensitivity or increased ability to discriminate between target present and target absent trials. Bias represents the tendency for an observer to respond “yes” to a target (negative *c* scores) or “no” (positive *c* scores) independent of their sensitivity.

## Experiments

### Experiment 1

#### Results

There is already data suggesting hands are a useful cue to an individual’s sex [32], [51]. Experiment 1 was designed to establish some of the parameters mediating sex discriminations from hand cues and to test for the existence of a male bias. More specifically, this experiment addressed three questions: When discriminating sex using hand cues (i) Is sex discrimination accuracy mediated by presentation duration? (ii) Is sex discrimination accuracy mediated by the availability of colour and texture cues? (iii) Are the effects of presentation duration and colour and texture information equivalent for both female and male hands? It was hypothesised sex discrimination performances would be best when colour and texture cues were present at longer presentation durations. It was also predicted that as stimulus ambiguity increased (when colour and texture cues were absent at short presentation durations) discrimination accuracy would, consistent with the existence of a male bias, decline more for female hands than for male hands.

**Figure 2:**
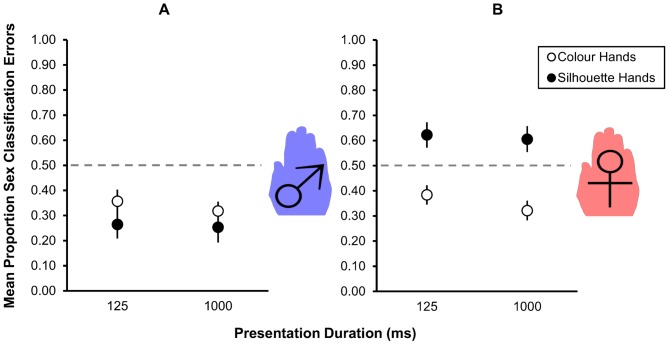
Sex classification error rates. Group proportions of sex classification errors in response to female (a) and male (b) hand images presented for 125 ms and for 1000 ms. Performances are shown for hands with colour/texture cues (open circles) and without (silhouettes: filled circles). Vertical bars represent ±1 SEM. Chance performance is represented by the dashed line. Performances above that line represent a systematic tendency to misreport stimulus sex.

Proportions of sex classification errors were calculated for each condition. An initial two-way mixed ANOVA was used to compare errors, across conditions, on the two absolute size conditions (small, large) and between female and male observers. There were no significant differences in error rates between the two sets of normalised hands (*F*_1,9_ = 0.28, *p* = .608), or between female and male observers (*F*_1,9_ = 0.64, *p* = .443), and no significant interaction between those variables (*F*_1,9_ = 0.83, *p* = .387). With that in mind, data were collapsed across those two variables and mean error rates calculated for each “hue/texture” condition (colour, silhouette), for each stimulus sex (female and male), at both presentation durations. Those means are shown in Figure 2.

As shown in Figure 2, when observers were presented with “coloured” hands (i.e. hue and texture preserved) error rates for female (panel B) and male (panel A) hands were similar within both presentation durations tested. Error rates for short presentation durations (female: 0.38±0.04; male: 0.36±0.05) were higher than the error rates observed at longer presentation durations (female: 0.32±0.04; male: 0.32±0.04). Nonetheless, performances on all those conditions were better than chance. These data confirm the findings reported by Kovács et al. [32]: Human observers can discriminate sex from hand cues alone and, with hue and texture cues available, can do so following just brief (125 ms) presentations.

When hue and texture cues were removed, performances on female and male hands diverged. Again, error rates at short presentation durations were higher than for longer presentation durations for female hands (125 ms: 0.62±0.05; 1000 ms: 0.61±0.04) and for male hands (125 ms: 0.27±0.05; 1000 ms: 0.26±0.06). Importantly, using the 2AFC paradigm employed here, error rates increased for judgements of female hands to levels greater than chance. Simultaneously error rates decreased for judgements of male hands when hue and texture cues were removed. Together these results suggest observers found sex discrimination using silhouette hands more difficult and, faced with ambiguity, shifted their response bias (see below) to increase their rate of “male” responding.

A set of four planned orthogonal contrasts tested the differences described above. There were significantly more errors made on short presentations than on long (*F*_1,10_ = 15.18, *p* = .002). Similarly, there were significantly more errors made on silhouette than on colour hands (*F*_1,10_ = 29.21, *p*<.001). Together, those two results suggest that hue and texture information and longer viewing times are important for accurate sex discrimination when using hand cues. Most importantly, while there were no differences between error rates on female and male hands when hue and texture cues were present (*F*_1,10_ = 0.05, *p* = .829), there was a significant difference between error rates on female and male silhouette hands (*F*_1,10_ = 13.03, *p* = .004). In other words, increasing ambiguity significantly changes the patterns of sex discrimination errors when using hand stimuli: Under conditions of high ambiguity, when hand sex cues are least salient, observers tend to report seeing male hands more often than female hands. Hue and texture seem to be critical for sex discrimination cues in the absence of information about absolute size. A summary of observer performance in Experiment 1 is available as Dataset S1.

#### Discussion

Experiment 1 was designed to investigate whether, when using hands as stimuli, (i) sex discrimination accuracy is mediated by presentation duration, (ii) sex discrimination accuracy is mediated by colour and texture cues, and (iii) those effects, if they exist, are equivalent for female and male hands. The data show that sex is discriminated more accurately at longer presentation durations and when colour and texture cues are available – conditions, in other words, under which sex cues are more clearly discernible. Interestingly, when cues were more ambiguous, performance on the sex discrimination task varied between female and male hands. Sex misclassification rates corresponding to female or male hands were respectively higher and lower than the 50% level expected if observers were guessing (Figure 2).

The systematically low error rates observed in the colour condition here are remarkable first of all because overt cultural sex cues (rings, nail polish, and so on) were unavailable to observers. It has been reported that infants as young as nine months of age can discriminate between adult female and male faces if sex-stereotyped cultural cues (e.g. clothing) are also visible [52]. Conversely, seven-year-old children could not visually distinguish sex from adult faces when cultural cues were minimised [37]. Clearly adult observers do not need such cues to discriminate sex at levels above chance. The low error rates in this experiment’s colour conditions are also exceptional because absolute hand size – a naturally dimorphic cue [51], [53] – was kept homogenous across stimuli. Thus, these data suggest that size is not a necessary cue and that hands can be used as an indicator of another’s sex, at least when colour and texture are available.

**Figure 3:**
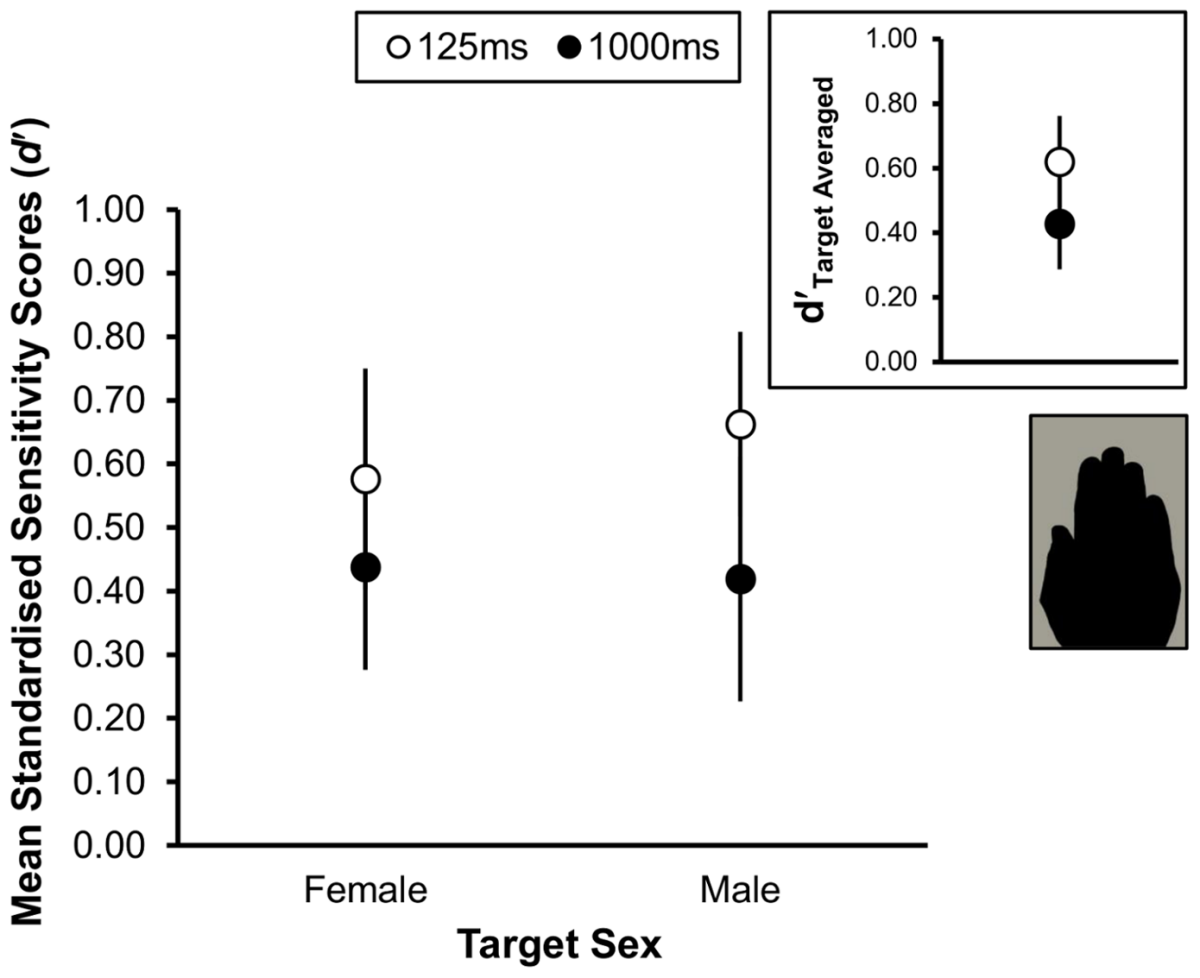
Sex classification sensitivity for ambiguous silhouette hands. Standardised group sensitivity (*d*′) scores, representing the ability to distinguish target from lure trials, as a function both of target sex (female and male) and whether hands were presented for 125 ms (open circles) or 1000 ms (filled circles). Data corresponding to silhouette hand stimuli conditions are depicted, although observers were also presented colour hands. Vertical bars represent ±1 SEM.

By comparison, performances in the absence of colour and texture cues diverged as a function of stimulus sex. Specifically, observers tended to categorise achromatic or silhouette hands more or less incorrectly depending on the hand’s sex. Female hand silhouettes were more often misjudged to be male. Male hand silhouettes were less often judged to be female (Figure 2). This result is, therefore, consistent with observations made previously using full-body and face-based stimulus sets [17], [18], [37], [38], [40] and may therefore represent a ubiquitous phenomenon. It is also, to the knowledge of these authors, the first demonstration of a male bias effect from hands that does not use either an adaptation or priming paradigm ([32] Figures 2(b) & 3 respectively).

What is not clear, from earlier reports or from this experiment, is the mechanism for that tendency. Experiment 2 was designed to explore the male bias observed in these data. Using a signal detection approach, we measured observer bias and sensitivity when discriminating sex from hand cues.

### Experiment 2

#### Results

Experiment 1 suggests that observers tend to report hands as looking “male” when cues that normally are sexually dimorphic are ambiguous. The aim of Experiment 2 was to explore the perceptual mechanisms mediating that bias in hands. In this experiment observers completed two signal detection tasks. In one, observers discriminated silhouette hands as female or not. In the other, observers discriminated silhouette hands as male or not. That technique makes it possible to discriminate whether the male bias manifests as the result of a difference in sensitivity to cues that signal female and male hands or from an observer bias.

It seems unlikely sensitivity differences will mediate the effect. For changes in sensitivity to be the cause, the variability of signal strength within whole-bodies, faces, and/or hands would need to differ as a function of stimulus sex. A more likely explanation for the male bias observed in Experiment 1 is a difference in observer bias. If observer bias is the mechanism driving the pattern of results reported here (and by inference in earlier studies), different response criteria should be observed for each target sex. More specifically, the pattern of responses observed in Experiment 1 can reflect either a conservative or strict criterion when assigning a target as female, a liberal or loose criterion when assigning a target as male, or by a combination of both.

Mean sensitivity performances for both target types at both presentation durations are shown in the main panel of Figure 3. Observers were able reliably to distinguish female targets from “noise”, and male targets from “noise” at both presentation durations. Most interestingly, when viewing silhouette hands observers were more sensitive at the shorter presentation duration (*d’*, female target = 0.58±0.17; male target = 0.66±0.15) than at the longer duration (*d*’, female target = 0.44±0.16; male target = 0.42±0.19). As shown in the inset panel of Figure 3, this trend persists when performance for female and male targets are averaged (*d*’, 125 ms = 0.62, ±0.14; 1000 ms = 0.43, ±0.14). Nonetheless, a post-hoc one-sample *t* test revealed that mean sensitivity at 1000 ms was greater than chance (*t*_10_ = 3.03, *p* = .013).

A one-way ANOVA shows there is no effect of stimulus sex (*F*_1,10_ = 0.04, *p* = 0.845), no effect of presentation duration (*F*_1,10_ = 3.37, *p* = 0.096), and no significant interaction between those factors (*F*_1,10_ = 0.48, *p* = 0.506) on sensitivity. In other words, there were no differences in observers’ sensitivities for discriminating female and male hand targets. The small decline in the sensitivity of observers across the two presentation durations was not significant here and neither did hand sex and presentation duration interact with each other in order to affect observers’ sensitivities. So, any effect of change in stimulus ambiguity on patterns of responding when discriminating female and male hands is not attributable to changes in sensitivity.

**Figure 4:**
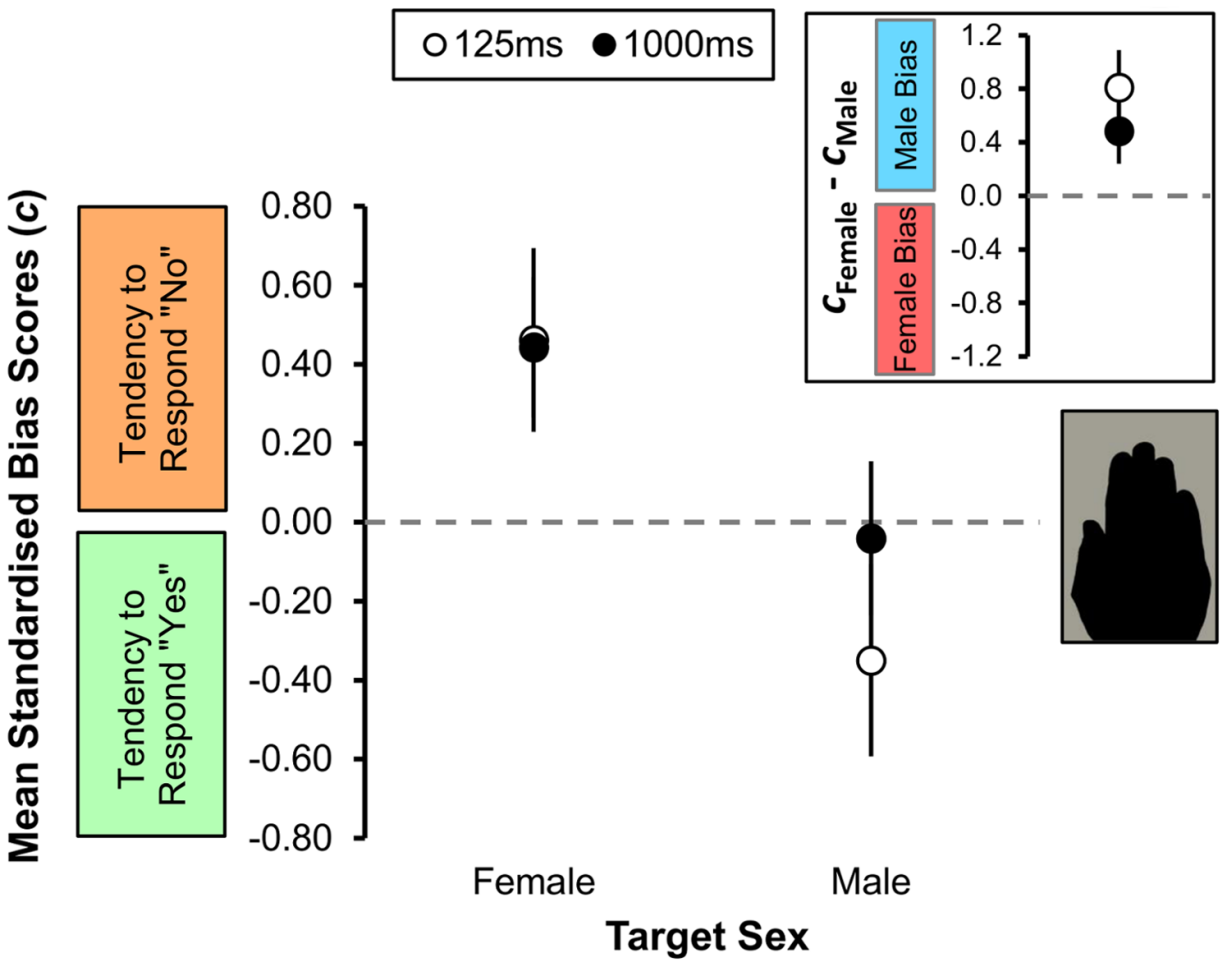
Sex classification bias. Standardised group criterion (*c*) scores, representing the tendency to respond ‘target absent’ (*c*>0) or ‘target present’ (*c*<0), as a function both of target sex (female and male) and presentation duration (125 ms: open circles; 1000 ms: filled circles). The insert shows the absolute mean bias score for both female and for male targets. Observers were generally male biased at both 125 ms (*c*_diff_ = 0.81±0.28) and 1000 ms (*c*_diff_ = −0.48±0.24) presentation durations. One-sample *t* tests indicated that the absolute mean bias was significant at the shorter (*t*_10_ = 2.92, *p* = .015), but not the longer (*t*_10_ = 1.99, *p* = .075) exposure time. Vertical bars represent ±1 SEM.

Observer bias does change, however, and those changes can explain the male bias observed in Experiment 1. As shown in the main panel of Figure 4, observers’ bias scores when searching for female targets was unaffected by presentation duration (125 ms: *c* = 0.46±0.23; 1000 ms: *c* = 0.44±0.18). Both scores are conservative in that they show a tendency to say “no” or target not present when searching for female targets. By comparison, presentation duration did affect mean bias scores when searching for male targets. Observers were more likely to say a target was male on short presentations (*c*, 125 ms = −0.35±0.24) than they were on longer presentations (*c*, 1000 ms = −0.04±0.20).

There was a significant difference in bias scores across the sexes such that observers were more conservative in judging female hands than male hands: Observers were more willing to say a target was “male” when searching for male targets than they were to say a target was “female” when searching for female targets (*F*_1,10_ = 6.62, *p* = .028). That pattern did not change as a function of presentation duration (*F*_1,10_ = 2.34, *p* = .157) but there was a significant interaction between target sex and presentation duration (*F*_1,10_ = 5.37, *p* = .043). That is, observers showed no change in bias when looking for female targets at either presentation duration (*c*, 125 ms = 0.46±0.23; *c*, 1000 ms = 0.44±0.18): Observers were consistently conservative in their attribution of a stimulus as a female target. By comparison, observers were liberal in their attributions of a stimulus as a male target. At 125 ms observers were most likely to say a stimulus was a male target (*c* = −0.35±0.24). As presentation duration increased to 1000 ms, observers became more conservative but were still more liberal in their target ascriptions than ever they were for females (*c* = −0.04±0.20). A summary of the sensitivity and bias data is available as supplementary material (see respectively Worksheet A & B, Dataset S2).

#### Discussion

The aim of Experiment 2 was to explore the perceptual mechanisms mediating the male bias observed in sex discriminations of ambiguous hands. The data reported here show observers were able reliably to distinguish female targets from “noise”, and male targets from “noise” both at short (125 ms) and long (1000 ms) presentation durations when viewing silhouette hands. Importantly, there were no differences in observers’ sensitivities for female and male hand targets. There were target sex differences in response biases however. Just as reported in Experiment 1 (using a 2AFC paradigm) the data in Experiment 2 (this time using a ‘yes/no’ signal detection paradigm) show the presence of a male bias. Under the most ambiguous conditions we tested, observers were conservative in their judgements of the presence of female targets, and that conservativism was not affected by presentation duration. When judging male hands, by comparison, observers were systematically liberal in their willingness to assign a target as “male” at shorter presentation durations, becoming less liberal as presentation duration increased.

These results confirm the reliability of hands as a sex cue available to observers, even in the absence of cultural and other features. More importantly though, they demonstrate the more specific perceptual mechanisms mediating the male bias. The sex-divergent response pattern described above suggests there are implicit differences in the cost/benefit analyses applied to the consequences of potential errors when searching for each target type. One possible interpretation of those differences is that the cost of a “miss” when searching for male targets is high compared to the cost of a false alarm [2], [4], [39]. Conversely, when searching for female targets the cost of a miss is lower perhaps than a false alarm. Whether the same ratios apply for sex discriminations from whole-body and from face cues needs next to be explored further, but seems likely given the apparent ubiquity of the male bias effect.

In summary, hands, like a number of other sexual dimorphisms (e.g. [17], [18], [37], [39]) elicit in observers a male bias when normally salient dimorphic cues are ambiguous. That bias is mediated both by conservative criteria for judging a target as female and liberal criteria for judging a target as male. The effect is not mediated by sensitivity differences and seems to be a real perceptual bias.

### General Discussion

The aim of the experiments reported here was to explore the proposal that there exists a multi-dimensional sex-perception space built around all available sexually dimorphic-s. One characteristic of such a space is that sex perceptions should arise not independently from cue-specific or even sense-specific processes, but should include also higher-order processes that are cue-independent. It was hypothesised that if a sex-perception space exists, the male bias that manifests during discriminations of other normally dimorphic cues [17], [18], [23], [38], [39], [40]–[42], [54] would also manifest for hands.

Experiment 1 established baseline performances for observers judging sex from human hands. The data show that observers could reliably discriminate an individual’s sex from their hands when colour and texture cues were present, even in the absence of absolute size cues. Performances deteriorated when presentation durations were shorter, and when colour and texture cues were removed. Nonetheless, the data were always consistent with observers making discriminations at levels not equal to chance. In particular, as stimulus ambiguity increased, sex discrimination performances diverged such that correct sex discriminations of female hands were fewer than for male hands. That is, the data reported here show evidence of a male bias when discriminating sex from ambiguous hand cues.

Experiment 2 explored the perceptual mechanisms mediating that bias. The data show that the effect does not arise from a difference in sensitivity. Instead, it arises – at least in this case of sexually ambiguous human hands – through a combination of a conservative criterion when judging targets as female and a liberal criterion when judging targets as male. The data also show that the criterion used for judging targets as female is relatively stable, while the criterion for judging targets as male is more labile.

The significance of that result lies in its implications for sex processing models. Should the same pattern of response biases eventually be shown to manifest for sex discriminations from other cues it will be strong evidence that sex discriminations are ultimately achieved via a higher-order process that is cue-independent. That would be good evidence for a multi-dimensional sex-perception space into which all cues contribute.

Already there is support that might be the case. Johnson et al. [39] found that categorisations of dimorphic cues were more often and more quickly ascribed as “male” unless the available cues were *exclusively* female. That is, the performances of Johnson’s categorisers were consistent with their applying very conservative criteria for discriminating stimuli as female and more liberal criteria for discriminating stimuli as male. Similarly, evidence exists for a male bias in auditory sex perceptions: Li et al. [54] examined the capacity for listeners to discriminate the sex of walkers from their footfalls. They reported observers performed the task reliably, but exhibited a tendency to identify as male the most ambiguous cases.

Neuroimaging data are also consistent. Podrebarac et al. [55] recently investigated sex discriminations using face stimuli. They found evidence that the left Fusiform Face Area (FFAl) preferentially changed its activity in response to sex but not identity repetitions. The FFA previously has been implicated in sex discriminations [56], [57] and it is likely that the FFA, probably on the left side, does contain sex-tuned neurons. In interpreting their data, Podrebarac et al. focussed on the complexity of sex discriminations and speculated that whilst FFA activation is necessary for sex discriminations, categorical sex judgements also recruit higher-level structures elsewhere in the brain. Interestingly, there is strong evidence that face-, body-, and hand-cues all mediate activity in the posterior Superior Temporal Sulcus (STSp) [58]–[66]. The STSp has been implicated too in the integration of biological visual and auditory cues [67], and is part of a larger network thought to mediate social perceptions [68], [69]. There is even evidence that STSp is involved in judging sex from faces [70]. If sex perceptions arise from processes that involve both the FFAl and the STSp, and no doubt other locations, it does seem likely that such perceptions are multi-dimensional. Todorov and colleagues [71], [72] have shown that in such spaces some dimensions can be orthogonal to each other, and some are not. Those relationships are yet to be mapped in the proposed sex-perception space.

One limitation of the data presented here is a lack of power to discriminate, if they exist, observer sex effects. There are reasons to expect that observer sex differences might be found in hand sex discriminations and in sensitivity to specific hand sex cues. Numbers of studies now are reporting reliable structural [73] and functional [74] differences between female and male brains and those differences carry over to performance differences in perceptual classification tasks [75]. It may be that such differences, if they exist, will be revealed as the specific sex cues mediating the effects reported here are unpacked. If so, it seems likely those effects will reflect a stimulus sex×observer sex interaction, with observers from each sex using different features to make their discriminations [76], [77].

## Conclusions

In summary, data from Experiments 1 and 2 suggest multiple key findings. The first is that hands are a useful sex cue, even when degraded. The second is that the male bias appears to be a ‘real’ effect, manifesting when normally sexually dimorphic cues are ambiguous and from different stimuli. The third is that the male bias manifests, at least for hands, from criterion differences between female and male cues: Observers apply a conservative criterion when judging cues as signalling female, and a liberal criterion when judging cues as signalling male. With other data these findings begin to build a picture of a multi-dimensional sex-perception space.

## Supporting Information

Dataset S1 **Sex classification error rates and summary of analyses (Experiment 1).**
(XLSX)

Dataset S2 **Sex classification sensitivity and bias scores and summary of analyses (Experiment 2).** The dataset contains two worksheets: Click the ‘Worksheet A’ tab to view a summary of the sensitivity data, and click the ‘Worksheet B’ tab to view a summary of the bias data.
(XLSX)
